# Criteria for designing integrated diagnosis interventions in low resource settings at the primary care level: a Delphi consensus study

**DOI:** 10.1186/s12913-025-13114-9

**Published:** 2025-08-25

**Authors:** Gamuchirai Pamela Gwaza, Annette Plüddemann, Marcy MacBain McCall, Sabine Dittrich, Carl Heneghan

**Affiliations:** 1 Department for Continuing Education, University of Oxford, Oxford, UK; 2 Centre for Evidence-Based Medicine, Nuffield Department of Primary Care Health Sciences, University of Oxford, Oxford, UK; 3 Department of Global Public Health, Deggendorf Institute of Technology, Deggendorf, Germany; 4 Department of Medicine, Centre for Tropical Medicine and Global Health, University of Oxford, Oxford, UK

**Keywords:** Africa, Delphi, Integrated diagnosis, Primary health care

## Abstract

**Background:**

Integrated diagnosis is crucial for addressing health challenges, particularly in managing comorbidities and chronic conditions. Technological advancements allow for rapid, simultaneous testing and diagnosis of multiple diseases. Integrated diagnosis interventions vary in purpose, models, diseases targeted, populations served, scale, and measured outcomes. This diversity, combined with varying levels of resource availability and health system capacity to respond effectively to positive diagnoses, makes it challenging to identify effective strategies. An effective integrated diagnosis approach can lead to early detection of critical and chronic conditions, improve patient experiences, and ultimately improve disease surveillance. This study aimed to establish core criteria for designing same-day integrated diagnosis interventions in primary care settings to enhance patient experiences and health outcomes in low- and middle-income countries (LMICs). The initial set of criteria was derived from a realist synthesis, which identified the key contextual factors and mechanisms required for such interventions to be effective.

**Methods:**

A two-round Delphi process engaged an international panel of fifty-five experts from diverse professions to establish consensus on core criteria for effective integrated diagnosis interventions. Predetermined consensus thresholds were set at 70% for *critical to include*.

**Results:**

A total of 55 experts participated in the first round. Participants represented various geographical regions, including Africa (*n* = 33), Europe (*n* = 17), Asia (*n* = 2), and the Americas (*n* = 2), and could be categorized into implementers (*n* = 36), policymakers (*n* = 7), and academics (*n* = 12). At the end of Round 1, fourteen of the thirty-three criteria reached a consensus as being *critical to include*, and nine criteria were removed. In Round 2, a total of 48 out of 55 experts participated and twelve criteria were considered. Four criteria reached a consensus as being *critical to include*. Through the two rounds of surveys, experts reached a consensus on 18 criteria.

**Conclusion:**

The study provides key criteria for prioritizing and developing integrated diagnosis interventions in primary care, in low-resource settings, particularly in Africa. The guidance might be invaluable for policymakers, funders, implementers, and manufacturers. The primary goal of integrated diagnosis is to enhance patient experiences and health outcomes. It is essential to consider all critical success factors during intervention design. These criteria may evolve as our understanding of integrated diagnosis advances.

**Supplementary Information:**

The online version contains supplementary material available at 10.1186/s12913-025-13114-9.

## Background

The diagnostic process includes performing a clinical history and interview, physical examination, diagnostic testing, and referring or consulting with other clinicians [1]. Integrated diagnosis, as it is conceptualized in this study, refers to identification and testing for multiple diseases or conditions during a single visit, with clients receiving the diagnosis on the same day [2]. This approach primarily targets the primary care level, emphasizing near or point-of-care diagnostics. In this case, integrated sample referrals, where patients receive the results on a different day, are excluded. An integrated diagnosis is a clinically essential intervention for some health conditions, such as preventing and treating tuberculosis (TB) co-infection with HIV [3]. Integrated diagnosis offers patients and clients increased convenience, improved access to healthcare services, continuity of care, and a holistic approach to addressing their health needs [2].

The health systems in low and middle-income countries (LMICs) are currently designed to respond to acute, predominantly infectious diseases, usually through disease-specific vertical programs that focus on a single condition/disease [4]. In Africa, where the burden of infectious diseases such as TB and HIV remains significant [5], the inability to accurately diagnose, treat, and identify missed cases hinders effective disease management. Furthermore, only 19% of patients have access to appropriate diagnostics at the primary healthcare level in LMICs, representing the single largest gap in the healthcare pathway [1, 6]. Primary care facilities, which play a crucial role in disease detection and management, often miss opportunities for preventing onward transmission. The World Health Organization (WHO) estimates, for example, that only 64% of new TB cases are detected and notified, suggesting that over three million cases of this highly contagious disease are missed annually [1, 7]. Moreover, as advancements in treatment continue to improve life expectancy and quality of life for people living with HIV, there is an emerging challenge of managing comorbidities with non-communicable diseases (NCDs), including cardiovascular disease, diabetes and cancers [8]. In addition, there is growing recognition of the intersection between chronic physical conditions and mental health disorders, which are often underdiagnosed and undertreated in this population. This shift necessitates a more comprehensive and integrated approach to care that goes beyond the traditional focus on infectious diseases to address the full spectrum of physical and mental health needs.

Diagnosis is just one step of the care pathway, and as such, integrated diagnosis functions as a specialized component within the broader framework of integrated healthcare. There is a high level of support for integrated health care in LMICs following the WHO policy on integrated health care in 2016 [9]. Diagnosis is mentioned as part of the health services, alongside health promotion, disease prevention, treatment, disease management, rehabilitation, and palliative care that should be integrated. Policy guidance is also available for integrating diagnosis and treatment of other health conditions such as TB and HIV [10].

Numerous well-intentioned interventions aimed at integrating healthcare service delivery at the point of care in primary care settings in LMICs often fall short of improving patient experiences and health outcomes despite increasing patient uptake [11, 12]. Regarding diagnosis, the tendency is to introduce diagnostic tools without fully considering other enabling aspects of the health system necessary for their success [2, 13, 14]. These aspects can encompass, for example, practical factors such as the electricity requirements of the instrument vis-a-vis the availability at the facility where it is placed, healthcare workforce capabilities, and treatment pathways, ultimately leading to suboptimal outcomes [14–16]. Moreover, there is pressure to put integration into policy mandates before implementation and feasibility are truly defined, creating a disconnect between policies and the realities of local health facility contexts. While some considerations may seem intuitive, there is a lack of clear and comprehensive guidance available to implementers and policymakers on how interventions for integrated diagnosis should be designed to enhance patient experiences and health outcomes.

Our objective was to expand upon existing studies in this field to bridge the significant gap in designing effective programs for integrated diagnosis. We aimed to identify key mechanisms and contextual factors crucial for the success of interventions. Additionally, we sought to systematically evaluate these criteria by establishing international consensus among implementers and policymakers. Our focus was on determining which criteria were deemed critical for inclusion when designing integrated diagnosis interventions aimed at improving patient experiences and health outcomes at the primary care level in low-resource settings, with a particular emphasis on Africa.

## Methods

This study used the Delphi method through an online survey administered to experts with knowledge and experience in integrated diagnosis. The Delphi is one method for improving the generation of critical ideas and the structured collection and processing of information gathered from experts [17]. Panels typically consist of 10 to 50 experts in the field, who are anonymous in that their identities are not disclosed to other panel members [18–20]. The Delphi method has been used extensively in developing criteria frameworks in the healthcare field [18, 21, 22].

Participants were purposefully sampled based on their knowledge and experience, The objective was to include a diverse group of experts across three main categories: implementers, policymakers or funders, and researchers or academic experts. The international expert group was identified as follows: (i) implementers- professionals currently working in healthcare in Africa, with a focus on integrated service delivery or diagnostics. This group included clinicians, nurses and laboratory specialist and other frontline providers; (ii) Policy makers or funders- individuals in decision-making roles within global health organizations or funding agencies such as WHO, Unitaid, Global Fund, and the Foundation for Innovative New Diagnostics (FIND), as well as technical managers and directors. This category also included health managers and policymakers from ministries of health based in African countries (iii) Researcher and academics- experts working in academic or research institutions with a focus on integrated healthcare or diagnostics. Many were recruited during the International Conference on Integrated Healthcare (ICIC-2023) in Belgium and have demonstrated expertise in the field. Efforts were made to ensure the participation of a wide variety of stakeholders in the study, focusing on geographic and occupational diversity within relevant areas of integrated health care and diagnostics and experts in various diseases and conditions (Table 1).

**Table 1:** Demographic details of participants in rounds 1 and 2

Characteristic		Round 1 (*n* = 55)	Round 2 (*n*−48)
Sex	Male	36% (*n* = 20)	35% (*n* = 17)
	Female	62% (*n* = 34)	65% (*n* = 31)
	No response	2% (*n* = 1)	-
Age	26–30	5% (*n* = 3)	-
	31–40	35% (*n* = 19)	29% (*n* = 14)
	41–50	36% (*n* = 20)	48% (*n* = 23)
	51–64	24% (*n* = 13)	23% (*n* = 11)
Region	Country where they are currently based		
Africa Round 1: 44% (*n* = 24) Round 2: 50% (*n* = 24)	Cameroon	1	1
	Ghana	1	1
	Ethiopia	1	1
	Kenya	1	1
	Lesotho	1	3
	Nigeria	1	1
	Rwanda	1	1
	Senegal	1	1
	South Africa	2	3
	Tanzania	1	1
	Uganda	1	1
	Zambia	2	1
	Zimbabwe	10	8
Europe Round 1: 42% (*n* = 20) Round 2: 38% (*n* = 18)	Belgium	2	1
	France	2	1
	Ireland	1	1
	Spain	1	1
	Switzerland	15	13
	United Kingdom	2	1
Asia Round 1: 7% (*n* = 4) Round 2: 6% (*n* = 3)	India	2	1
	Nepal	1	1
	Singapore	1	1
United States 4% (*n* = 2) both rounds	The Americas	2	2
N/A 4% (*n* = 2)	No response	2	2
Profession			
Academic Researcher/Graduate Student	Scientist/Researcher	11% (*n* = 6)	13% (*n* = 6)
	Professor/Lecturer	7% (*n* = 4)	-
	Graduate student	4% (*n* = 2)	2% (*n* = 1)
Health Policymaker (*n* = 13)	Senior government officials	11% (*n* = 6)	8% (*n* = 4)
	Management and leadership in global health organization	13% (*n* = 7)	15% (*n* = 7)
Implementer	Professional working in a global health organization, e.g., program manager/officer	27% (*n* = 15)	31% (*n* = 15)
	Medical laboratory scientist	11% (*n* = 6)	13% (*n* = 6)
	Medical doctor/Specialist physician	9% (*n* = 5)	10% (*n* = 5)
	Data scientists/Surveillance/technical officers	7% (*n* = 4)	8% (*n* = 4)

Understanding that some experts may be experts in more than one domain, email invitations were sent to 107 experts. The target was to obtain at least 50 experts in the study from the larger sample. None who responded were excluded.

This study built on a previous realist review on integrated diagnosis in LMICs which identified the key contextual factors and mechanisms required for such interventions to be effective [2]. All the criteria identified in the review were included in the initial list for prioritisation. These insights formed the basis of the criteria that were selected for prioritization. There was a total of 33 criteria divided into six domains, each comprising specific criteria: Governance (8 criteria), Operational Considerations (5 criteria), Physical Integration (8 criteria), Human Resources Integration (4 criteria), Technology Integration (4 criteria) and Monitoring and Evaluation (M&E) (4 criteria). See Supplementary file 1. The survey was piloted with 4 people; two laboratory technicians who implemented integrated diagnosis interventions, a health planner and primary care provider and was refined based on the comments received.

Two rounds of surveys were administered online using the Jisc platform, a digital solution, mostly targeting UK education and research, including tools and services for data collection, learning technologies, and surveys. Respondents rated each criterion using a 1–5 Likert scale with 1-*Not important*, 2-*limited importance*, 3-*important but not critical*, 4- *critical to include* and 5- *Unable to rate* option should they feel that item to be beyond their expertise. ‘Consensus’ was considered achieved if 70% or more of the participants rated criteria four or *Critical to Include* when assessing significance. Consensus proportions for each question were determined based on the number of respondents, excluding those who selected category 5-*Unable to rate (Not my expertise)*. Consequently, the denominator for calculating consensus comprised only participants possessing relevant knowledge and expertise in the specific question under consideration.

In the Round 2 survey, criteria that previously received votes ranging from 50 to 69% for being *critical to include* [4] were all included. This approach was taken unless there were specific instances where criteria were merged or rephrased, and explanations would be provided. The intention was to include criteria in the Round 2 survey that had garnered significant support in terms of importance but had not reached the threshold for consensus in Round 1. Additional criteria could be included in Round 2 based on recommendations by the participants. Criteria that had received less than 50% for being *critical to include* [4] were removed and not included in the final criteria after Round 1.

## Results

In the first round, 55 experts participated, representing diverse geographical regions: 44% from Africa, 42% from Europe, 7% from Asia, and 4% from the Americas, with 4% not indicating their location. Most survey respondents were based in Switzerland (*n* = 15), predominantly comprising international staff from prominent global health organizations such as FIND, WHO, Unitaid, and the Global Fund. Additionally, there was a notable number from Zimbabwe (*n* = 10), the researcher’s home country, as they leveraged personal networks and word of mouth to engage more implementers and policymakers. Among the participants, 54% were healthcare implementers, 24% were academics, and 22% were policymakers.

In Round 2, 48 out of the original 55 participants responded. 65% were female and 35% were male. Geographically, 50% were based in Africa, 38% in Europe, 6% in Asia, 4% in the Americas, and 2% did not identify their location. In terms of professional roles, 63% were implementers, 23% were policymakers and 15% were academics and researchers (Table 1).

### Round 1

Fourteen criteria achieved consensus, with 70% or more participants considering them as Category 4, “critical to include”, while ten criteria were eligible to proceed to Round 2. Table 2 below displays the percentage of votes for each criterion in Round 1.

**Table 2:** Total results of round 1

		Critical to include (4)	Important but not critical (3)	Limited importance (2)	Not important (1)
	Domain 1: Governance				
1	Clear and specific funding for diagnosis and treatment	81% (*n* = 44/55)	13% (*n* = 7)	4% (*n* = 2)	2% (*n* = 1)
2	Funding for continued training of the healthcare workers for diagnosis	74% (*n* = 40)	20% (*n* = 11)	6% (*n* = 3)	0
3	System for coordination of donor support to avoid disease fragmentation	59% (*n* = 32)	39% (*n* = 21)	2% (*n* = 1)	0
4	WHO policy or recommendation for integration	33% (*n* = 18)	55% (*n* = 30)	13% (*n* = 7)	0
5	National policy or guideline for integration	85% (*n* = 47)	13% (*n* = 7)	2% (*n* = 1)	0
6	Diagnostic algorithm or screening tool to guide diagnosis	87% (*n* = 48)	13% (*n* = 7)		0
7	Treatment or clear referral pathway	91% (*n* = 49)	7% (*n* = 4)	2% (*n* = 1)	0
8	Strong leadership, with shared vision and support for integration	81% (*n* = 44)	17% (*n* = 9)	2% (*n* = 1)	0
	Domain 2: Operational Considerations				
9	Complexity of diagnosis for diseases integrated	68% (*n* = 36)	28% (*n* = 15)	4% (*n* = 2)	0
10	Similar prevalence of diseases included	6% (*n* = 3)	33% (*n* = 18)	43% (*n* = 23)	19% (*n* = 10)
11	Similar target population or risk profile of intended beneficiaries	22% (*n* = 12)	52% (*n* = 28)	19% (*n* = 10)	7% (*n* = 7)
12	Combined clinical & ICT systems, including a single registration form	58% (*n* = 30)	37% (*n* = 19)	4% (*n* = 2)	2% (*n* = 1)
13	Healthcare worker training in patient-centred care	74% (*n* = 40)	20% (*n* = 11)	4% (*n* = 2)	2% (*n* = 1)
	Domain 3: Physical Integration				
14	Sufficient physical space for private examinations	72% (*n* = 38)	26% (*n* = 14)	2% (*n* = 1)	0
15	Sufficient and comfortable space to sit while patients wait their turn	43% (*n* = 23)	48% (*n* = 26)	9% (*n* = 5)	0
16	Clear directions on how to navigate the facility to access different services	67% (*n* = 36)	30% (*n* = 16)	4% (*n* = 2)	0
17	Healthcare worker training in interpersonal collaboration	72% (*n* = 39)	26% (*n* = 14)	0	2% (*n* = 1)
18	Trust and respect among healthcare workers s of each other’s expertise	54% (*n* = 29)	41% (*n* = 22)	4% (*n* = 2)	2% (*n* = 1)
19	Clear roles assigned to each healthcare worker at the facility	87% (*n* = 47)	7% (*n* = 4)	6% (*n* = 3)	0
20	Functional referral mechanism to access other services	84% (*n* = 46)	11% (*n* = 6)	2% (*n* = 1)	4%
21	Follow-up mechanisms of patients who need to access referral services	76% (*n* = 41)	19% (*n* = 10)	4% (*n* = 2)	2% (*n* = 1)
	Domain 4: Human Resource Integration				
22	Training in diagnosis and follow-up counselling	89% (*n* = 49)	9% (*n* = 5)	2% (*n* = 1)	0
23	Healthcare workers should initiate diagnosis rather than the patient requesting it	52% (*n* = 26)	42% (*n* = 21)	4% (*n* = 2)	2% (*n* = 1)
24	Plan of how long it takes on average to conduct the diagnosis	47% (*n* = 25)	49% (*n* = 26)	2% (*n* = 1)	2% (*n* = 1)
25	Continuity of care: one healthcare worker manages the health care of the same patient	28% (*n* = 15)	39% (*n* = 21)	30% (*n* = 16)	4% (*n* = 2)
	Domain 5: Technology Integration				
26	All technical conditions for the diagnostic device, screened at the point of care, to function are met	89% (*n* = 48)	11% (*n* = 6)		
27	The test can be used as a screening tool; another test can confirm the results	33% (*n* = 18)	44% (*n* = 24)	20% (*n* = 11)	2% (*n* = 1)
28	The test should be both a screening and confirmatory tool for the results.	30% (*n* = 16)	45% (*n* = 24)	19% (*n* = 10)	6% (*n* = 3)
29	Healthcare workers should be able to conduct the test alone without other HCWs	33% (*n* = 17)	42% (*n* = 22)	15% (*n* = 8)	23% (*n* = 5)
	Domain 6: Monitoring and Evaluation (M&E)				
30	An M&E framework with measurable indicators for intended outcomes	61% (*n* = 33)	33% (*n* = 18)	4% (*n* = 2)	2% (*n* = 1)
31	A reporting requirement for healthcare workers to provide reports on all diseases	57% (*n* = 31)	31% (*n* = 17)	6% (*n* = 3)	6% (*n* = 3)
32	Single reporting form or tool at the facility for all the targeted diseases	58% (*n* = 31)	34% (*n* = 18)	8% (*n* = 4)	0
33	Customer feedback mechanism to report on the quality of service	54% (*n* = 29)	41% (*n* = 22)	4% (*n* = 2)	2% (*n* = 1)

Feedback was offered concerning specific criteria in each domain, with some recommendations for rephrasing and adding new criteria for consideration. There were no discernible patterns of voting based on demographic factors.

## Domain 1: Governance

Consensus was reached for six out of eight criteria as being “*critical to include”*. There were additional comments to support the funding criteria, which achieved consensus from two implementers, drawing on experiences from African countries who emphasized the preference for funding that cuts across diseases rather than being compartmentalized, especially in conjunction with historically disease-specific funding. Furthermore, another implementer commented that the available funding information should be made available at the service delivery level, as often, this information is only available at the national decision-making level.

The criterion on a system for coordinating donor support to prevent disease fragmentation (59%), had votes above 50% in category 4, “*critical to include”*, and proceeded to Round 2.

However, the other criterion regarding WHO policy or recommendation for integration (33%) received less than 50% support and was subsequently removed. One comment from an implementer in a global health organization based in Europe was that integration of diseases, whether at the national or global policy level, does not inherently lead to integrated diagnosis. They argued that successful integration depends on the availability of funding and the presence of implementation setups capable of facilitating it at a country level.

There were three additional criteria recommended to be added by different experts. The first from an implementer was to add disease management to treatment pathways, given that certain diseases, such as NCDs, require effective management rather than curative measures, and treatment could suggest curable treatment. The second criterion added was from a policymaker to add policies enabling the full utilization of diagnostic tools at the primary care level. In some countries, not all health workers are certified to conduct all diagnoses, such as blood-based tests, and they need the necessary approval from the government. These two recommendations were added in Round 2. The third recommendation from an implementer based in an African country was to establish a government agency responsible for the supervision, regulation, and standardization of clinical and medical laboratories. The recommendation was not included in Round 2, as it was deemed to be too focused on laboratories, which were not always available at the primary care level, the main target of the study.

## Domain 2: Operational considerations

One out of the five criteria achieved consensus on the training of healthcare workers in patient-centered care. Two criteria had votes above 50% in Category 4 “*critical to include”* and were moved to Round 2. These were the complexity of diagnosis and combined clinical and ICT systems, including a single registration.

Additional comments were received for the two criteria with above 50% votes. Regarding the complexity of diagnosis, two implementers offered different comments. The first was that there was a need to strike a balance between diagnostic complexity and the need for innovative approaches that streamline diagnostic procedures at the primary care level. The second comment was that the operational functionality and acceptability of the diagnostic algorithm at the facility level outweigh the simplicity of the test itself; thus, the ability and ease of integrating the diagnostic into existing procedures is more important than whether it is complex. This criterion was rephrased to reflect this perspective in Round 2. The criterion specifying combined clinical and ICT systems, including a single registration form, was suggested to be rephrased as integrated information systems in Round 2. The adjustment would allow for a broader interpretation that could encompass elements discussed in Domain 6 related to monitoring and evaluation. The suggestion was adopted.

Two criteria had below 50% votes and were removed. Although there was no consensus that a similar target population or risk profile approach was critical, there was a comment that the approach could effectively address urgent needs in outbreak scenarios. However, mechanisms for integrating post-outbreak surveillance into routine services were deemed essential for sustainability and scalability.

There were recommendations to add three criteria to this domain, proposed by three different implementers, (i) Community awareness, demand, and acceptability of the diagnostic services under consideration; (ii) Availability of medical insurance or schemes to assist with out-of-pocket expenditures for services; (iii) internet connectivity. The first two recommendations were adopted. However, the third was not included as internet connectivity could be included under other criteria in the technical integration domain.

## Domain 3: Physical integration

Five out of the eight criteria reached a consensus. Although it achieved consensus, there was a comment from an implementer that a functional referral mechanism could only work if there was also an effective follow-up mechanism to track adherence and loss in the diagnostic pathway. This was added in Round 2.

The two criteria received over 50% support and advanced to the second round. These criteria were clear directions on how to navigate the facility to access different services (67%), and trust and respect among healthcare workers for each other’s expertise (54%). There was a comment that clear directions on how to navigate the facility may not be sufficient without establishing clear patient pathways and points of contact to guide patients through different services. This criterion was refined in Round 2 to reflect this. Secondly, the criterion on the importance of trust and respect among HCWs for each other’s expertise was acknowledged. Still, it was noted to be challenging to understand or monitor this social and behavioral aspect at the facility.

However, one criterion, sufficient and comfortable space for patients to sit while waiting their turn (43%), did not achieve consensus and was subsequently removed. An implementer based in Africa commented on this criterion that the requirement for sufficient and comfortable waiting space needed to be revised for low-resource settings, which may face constraints in terms of space and budget.

There were suggestions to add some criteria to this domain: (i) Deliberate investment to increase the number of trained health workers, including nurses, doctors, and laboratory scientists; (ii) Adequate incentives for healthcare workers, such as continuous training. These were not added as they were assumed to be included under the funding in domain 1.

## Domain 4: Individual or human resource integration

A consensus was achieved for one out of the four criteria, which was HCW training on diagnosis and follow-up counseling. Although the criterion on training achieved consensus, there was a comment by a policymaker based in Africa that the training needed to be qualified. They were of the view that many training programs did not result in improved competence and ability to apply the knowledge and skills acquired effectively.

One criterion had over 50% votes: HCW should initiate diagnosis rather than the patient requesting it (52%) and was included in Round 2. There were comments on this criterion by three implementers. Firstly, it was highlighted that care and respect must be maintained throughout the diagnostic process. Secondly, it was emphasized that providers should engage in a dialogue with patients rather than dictating the diagnosis process. Thirdly, considerations were noted regarding costs that health workers may not always consider when recommending or referring to diagnostic tests. It was suggested that minimum criteria be set for providers as to when initiating integrated diagnosis might be necessary.

The remaining two criteria did not achieve consensus and were removed. The criteria for continuity of care were recommended to be broadened. In LMICs, continuity of care is not just about individualized care but can also be achieved by establishing a robust referral system that is well integrated into the healthcare framework. Functional referral systems can ensure a seamless transition of patients between different levels of care and contribute to overall healthcare integration.

## Domain 5: Technical integration

Consensus was achieved for one of the four criteria, which was the technical conditions for the instrument to function are met.

However, a consensus was not reached on the remaining criteria, which were whether the test was a screening or confirmatory tool, or both, and whether the healthcare worker could conduct the test alone without assistance from other healthcare workers. As a result, these criteria were removed from consideration. Five implementers provided feedback on these criteria. They noted that whether the tool served as a screening or confirmatory measure or whether the health worker could independently conduct the test depended on context. Factors such as disease prevalence and the specific setting, facility, or diagnostic tool in use influenced these considerations, making them unsuitable as core criteria. Instead, emphasis was placed on healthcare workers being knowledgeable about the diagnostic algorithm for various diseases, whether they could conduct the test themselves or refer patients accordingly.

There were proposals to include additional criteria related to basic infrastructure for ensuring the security of diagnostic platforms and related consumables, as well as adherence to process safety management standards, and biosafety and bio-security elements. These were not added in Round 2 as they could be addressed within the broader context of the technical requirements of the diagnostic tool.

## Domain 6: Monitoring and evaluation (M&E)

None of the four criteria gained consensus. However, all four criteria were above the 50% threshold and were included in round 2. There were some comments from two implementers who indicated concerns about the additional burdens placed on already overworked healthcare workers due to reporting requirements from various donor agencies. There was also a perspective suggesting that customer feedback on the quality of care might be considered a separate objective from integration. The argument put forth was that integrated diagnosis should focus on a multi-disease testing approach in laboratories or clinics rather than on the patient-centered approach. A decision was made to merge some of the criteria in domain six into two criteria instead of four. For example, customer feedback was included as part of the M&E instead of a separate criterion.

At the end of Round 1, twelve criteria were moved to Round 2; three of them were new additions, as indicated in Table 3 below.

**Table 3:** Results of round 2 survey

		Critical to include	Important but not critical	Limited importance	Not important	Total
	Domain 1: Governance					
1	System for coordination of donor support to avoid disease fragmentation	77% (*n* = 36)	23% (*n* = 11)			100%
2	Policies to allow full use of diagnostics at the primary healthcare level, e.g., necessary certification for healthcare workers or government allowance. (NEW)	68% (*n* = 32)	28% (*n* = 13)	4% (*n* = 2)		100%
	Domain 2: Operational Considerations					
3	Operational functionality and acceptability of the diagnostic algorithm	79% (*n* = 37)	21% (*n* = 10)			100%
4	Integrated information systems, including a single registration form	50% (*n* = 23)	46% (*n* = 21)	4% (*n* = 2)		100%
5	Community awareness, demand and acceptability of the diagnostic services being considered (NEW)	70% (*n* = 33)	23% (*n* = 11)	4% (*n* = 2)	2% (*n* = 1)	100%
6	Availability of medical insurance or medical schemes to help with out-of-pocket expenditure for services for diagnostic services (NEW)	45% (*n* = 21)	36% (*n* = 17)	17% (*n* = 18)	2% (*n* = 1)	100%
	Domain 3: Physical Integration					
7	Clear patient pathway and point of contact to guide the patient through different services	83% (*n* = 38)	15% (*n* = 7)	2% (*n* = 1)		100%
8	Trust and respect among healthcare workers of each other’s expertise	44% (*n* = 21)	54% (*n* = 26)	2% (*n* = 1)		
	Domain 4: Human Resource Integration					
9	Healthcare workers should initiate diagnosis rather than the patient requesting it	40% (*n* = 19)	46% (*n* = 22)	15% (*n* = 7)		100%
	Domain 6: Monitoring and Evaluation (M&E)					
10	An M&E framework with measurable indicators results	55% (*n* = 24)	43% (*n* = 19)	2% (*n* = 1)		100%
11	A single national-level health information system for reporting, which includes all the relevant diseases and conditions	66% (*n* = 31)	32% (*n* = 15)	2% (*n* = 1)		100%

### Round 2

Four criteria achieved consensus as “*critical to include*,” with 70% or more agreement (Table 3). One criterion falls under the governance domain, emphasizing the importance of a system of donor coordination to prevent disease fragmentation. The other two criteria, both within the operational consideration domain, focus on the operational functionality and acceptability of the diagnostic algorithm as well as the community awareness, demand, and acceptability of the diagnostic services, highlighting its significance. The fourth criterion in domain 3 on Physical integration is a clear patient pathway and point of contact to guide the patient through different services.

Four criteria received between 50% and 69% agreement, indicating they did not meet the consensus threshold for being deemed “*critical to include*” but were still considered important. The first criterion focuses on policies allowing the full use of diagnostics at the primary care level.

Three of these criteria relate to M&E:

1. The need for integrated information systems, including a single registration form.
2. The importance of having an M&E framework with measurable indicators and results.
3. The necessity for a single national-level health information system for reporting encompasses all relevant diseases and conditions.

There were no additional comments on the criteria.

Based on the Delphi results, eighteen criteria were identified as core criteria to be prioritized when designing integrated diagnosis interventions that result in optimum patient experiences and health outcomes. See Table 4 for the full list of core criteria that reached a consensus.

**Table 4:** Core criteria that reached consensus

	Criteria	Critical to include
	Domain 1: Governance	
1	Treatment/disease management or clear referral pathway	91%
2	Diagnostic algorithm or screening tool to guide diagnosis	87%
3	National policy or guideline for integration	85%
4	Strong leadership, with shared vision and support for integration	81%
5	Clear and specific funding for diagnosis and treatment	81%
6	System for coordination of donor support to avoid disease fragmentation	77%
7	Funding for continued training of the healthcare workers for diagnosis	74%
	Domain 2: Operational considerations	
8	Operational functionality and acceptability of the diagnostic algorithm	79%
9	Healthcare workers training in patient-centered care	74%
10	Community awareness, demand, and acceptability of the diagnostic services being considered	70%
	Domain 3: Physical integration	
11	Clear roles assigned to each healthcare worker at the facility	87%
12	Functional referral mechanism to access other services	84%
13	Clear patient pathway and point of contact to guide the patient through different services	83%
14	Follow-up mechanisms of patients who need to access referral services	76%
15	Sufficient physical space for private examinations	72%
16	HCW training in interpersonal collaboration	72%
	Domain 4: Human resource integration	
17	Training in diagnosis and follow-up counseling	89%
	Domain 5: Technical integration	
18	All Technical conditions for the diagnostic device, screened at the point of care, to function are met	89%

## Discussion

The Delphi study aimed to prioritize factors that were essential when designing integrated diagnosis interventions at the primary care level in low-resource settings where trade-offs must be made constantly. Eighteen core criteria across five domains were identified All of the 18 criteria can be aligned with the WHO health systems framework [23], predominantly situated within the leadership/governance and service delivery pillars (Table 5).

**Table 5:** Mapping the core criteria to the WHO health systems framework

WHO Health Systems Framework	Core criteria for integrated diagnosis interventions	Domain in the Delphi study
Leadership/Governance	• A system to coordinate donors • Strong leadership, with shared vision and support for integration • National policy or guidelines for integration	Domain 1: Governance
Financing	• Clear and specific funding • Funding for training	Domain 1: Governance
Health workforce	• Training on patient-centered care • Training on diagnosis and follow-up counseling, • Training on interpersonal collaboration	Domain 2: Operational Considerations Domain 3: Physical integration Domain 4: Human Resource Integration
Service delivery	• Diagnostic algorithms or screening tools for integration • Operational functionality of diagnostic algorithms • Community awareness, demand, and acceptability of diagnostics • Sufficient physical space for private examinations • Clear roles assigned to each HCW at the facility. • Functional referral mechanisms to access other diagnostic services. • Clear patient pathway to guide the patient through different services. • Follow-up mechanisms of patients who need to access referral services	Domain 1: Governance Domain 2: Operational Considerations Domain 3: Physical integration
Health products & technologies	• All Technical conditions for the diagnostic device, screened at the point of care, to function are met • Treatment or clear referral pathway	Domain 5: Technical Integration Domain 1: Governance
Health Information systems	No criteria matching that reached a consensus	

The Leadership/Governance pillar aligns with Domain 1 (Governance), encompassing criteria such donor coordination mechanism, strong supportive leadership, and the presence of national integration policies and guidelines. Prioritizing these aspects reflects the need for structural integration, which underpins successful implementation.

Within the service delivery pillar, most criteria fall under Domain 2 and 3 (Operational considerations and Physical integration). Operational factors, such as energy and temperature requirements, internet access, refrigeration requirements, supply chain reliability and facility infrastructure must be assessed. For example, diagnostics tools may end up lying idle and unused in health facilities due to electricity constraints or difficulty souring spare parts [15, 24, 25]. Facility layout also affects service integration, particularly for stigmatized conditions, where patient privacy and navigation pathways are essential to a positive care experience. Unfortunately, patient perspectives are often overlooked, leading to underutilization of resources. Addressing these practical considerations and engaging communities in service design is crucial. Increasingly, participatory approaches are recognized as key to demand generation and service uptake, for example in the introduction of self-testing technologies [26, 27].

The health products and technologies pillar align domain 5 (Technical integration). Ensuring diagnostic tools meet technical and contextual requirements, such as the WHO’s REASSURED criteria, is essential for their adoption and use [28, 29]. However, the introduction of new diagnostics does not always lead to their optimal use. In some cases, tests are added rather than substituted [1, 15, 30]. Global health organizations like Unitaid are increasingly addressing these challenges by supporting the development and deployment of tools tailored to LMIC settings and integrated within local diagnostic algorithms [31, 32].

The health workforce pillar is reflected in Domain 4 (Human resource integration) related to HCW training, interpersonal collaboration, and patient-centred care. Improving integrated diagnosis services depends not only on what is delivered but also who delivers it and how. Training efforts should balance technical skills with interpersonal and collaborative competencies. While structural integration has received more attention in the literature, growing evidence highlights the significance of social dimensions—such as teamwork and communication—in shaping both patient and provider experiences, as well as perceptions of quality and integration [33, 34].

Notably, no criteria achieved consensus within the Health Information Systems pillar. Criteria such as the implementation of an M&E framework, integration of disease-specific data systems, and the use of unified patient registration forms were considered important but not critical, receiving between 50% and 69% of votes. This suggests a perceived lower priority for information system integration in the immediate context of designing effective diagnostic interventions.

### From criteria to implementation

The diversity of prioritized criteria highlights the need for a holistic approach when introducing integrated diagnostic interventions in primary healthcare facilities. While individual criteria such as expanding diagnostic algorithms or implementing dual testing may increase coverage and reduce costs, they do not necessarily translate into better health outcomes or patient experiences [35].

Diagnosis functions as part of a broader system. Changes in one area inevitably affect others. Improvements in diagnostic technologies alone do not guarantee improvements in patient care [30]. Assessments of diagnostics often focus on test performance, overlooking contextual factors such as facility infrastructure, patient pathways, and health system readiness. As a result, policymakers may favour cost over diagnostic quality or downstream impact, ultimately limiting the effectiveness of interventions.

However, several barriers may hinder the implementation of integrated diagnostics in low-resource primary care settings, as already highlighted in the criteria. These include limited infrastructure (e.g., unreliable electricity, insufficient space for confidential testing), shortages of trained personnel, fragmented care pathways, and weak referral and follow-up systems. In addition, resistance to change among healthcare workers or lack of alignment with national policies can impede adoption.

To address these challenges, implementation efforts must be tailored to the local context. This includes engaging frontline providers in intervention design and ensuring adequate training not only in diagnostic procedures but also in patient-centred care and interdisciplinary collaboration. Treatment pathways and disease management must be embedded within the diagnostic strategy- either within the facility or through functional referral mechanisms with appropriate follow-up. Effective implementation requires comprehensive “deployment packages” that are tailored to existing structures and patient care processes [25].

**Fig. 1:**
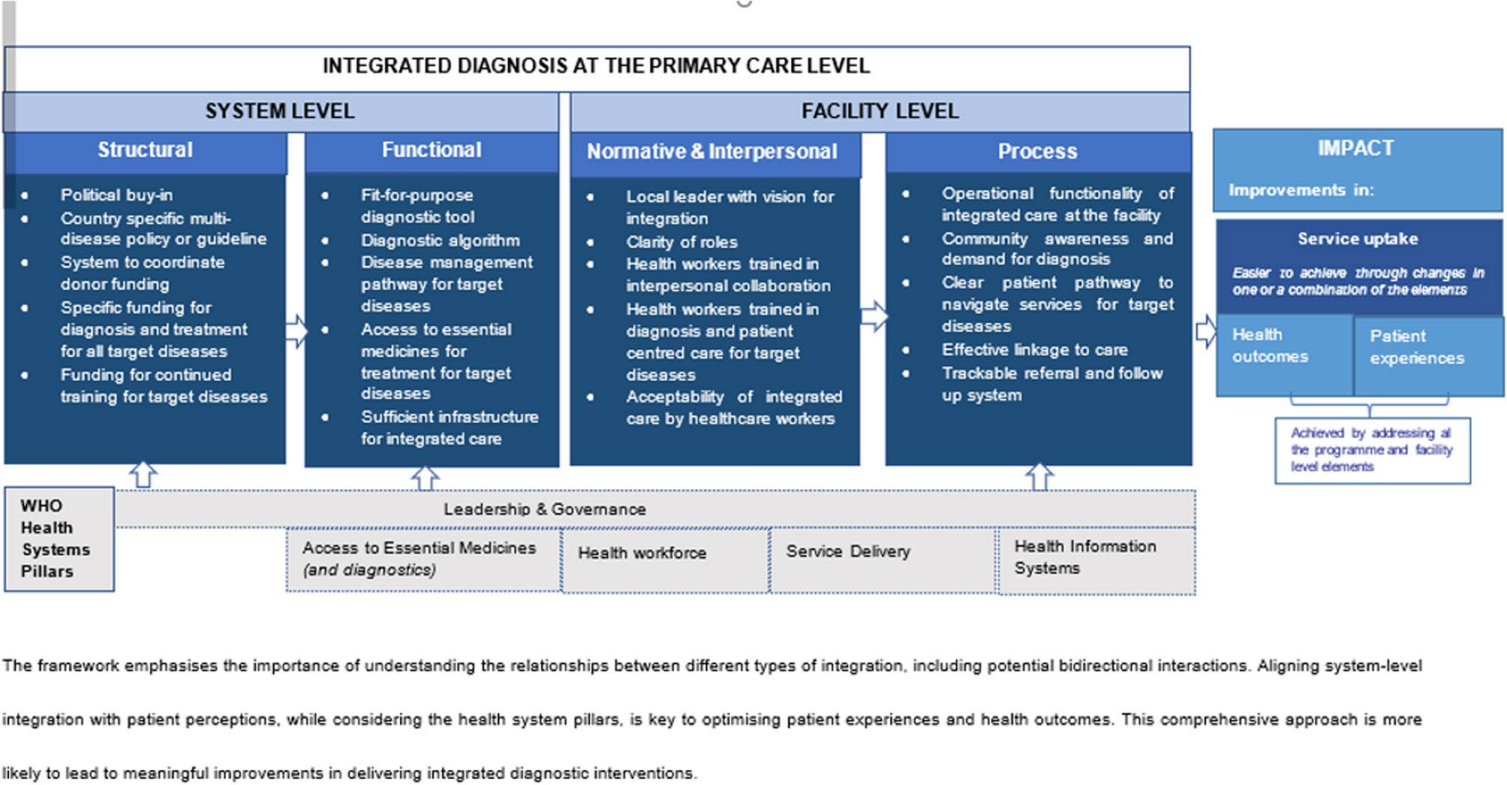
Conceptual framework for effective same-day integrated diagnosis interventions at the primary healthcare level in low-income settings

Figure 1 presents a proposed framework that encapsulates this holistic perspective. It aligns the prioritized criteria with the WHO health systems building blocks and draws on key principles from integration theory. Integration theory identifies five dimensions of healthcare integration: structural and functional integration at the system level, and process, normative, and interpersonal integration at the facility level. These dimensions correspond closely with the identified criteria and help clarify the level at which each criterion is best applied. Structural integration refers to shared infrastructure, financial arrangements, or centralized management systems. Functional integration involves coordinated protocols and operational procedures. Process integration focuses on service workflows, while normative and interpersonal integration relate to shared values, collaboration, and teamwork among healthcare providers.

### Limitations

While valuable, the Delphi methodology has acknowledged limitations in representativeness [17, 36, 37], particularly since the selected experts may hold specific viewpoints or agendas, limiting the generalizability of the findings. For instance, the lack of consensus on the M&E domain in this study might reflect the backgrounds of experts not working in M&E or appreciating its value.

The Delphi approach does not rely on a large sample size for generating robust insights [17, 38]. This study aimed to gather input from a diverse range of experts from industry, academia, government, and health services to ensure a broad spectrum of perspectives. The 55 experts who participated were based in 23 different countries and had relevant working experience across an additional 59 countries in the health sector, specifically related to diagnosis or integrated health care. They also represented a mix of professional roles, including as implementers, policymakers, or researchers, and brought substantial experience from LMICs, particularly in Africa, where the study is most relevant. As such, the core criteria presented reflect a rich diversity of viewpoints and are not grounded in any single healthcare system, profession or geographic context.

However, as with any Delphi study, non-response may have introduced some degree of selection bias. While the overall sample appeared balanced in terms of geography and professional background, it is possible that certain perspectives—particularly from individuals with more limited engagement in integrated diagnostic initiatives or from underrepresented regions—may not be fully captured. Unfortunately, we were unable to systematically characterize non-respondents due to the nature of the recruitment and participation process. Future studies could explore more targeted strategies to ensure the inclusion of a wider range of viewpoints, particularly from settings or constituencies that may have been underrepresented in this round.

Despite these limitations, Delphi studies remain a valuable method for achieving consensus in situations where face-to-face interactions are impractical, and the collective judgment of experts is essential.

## Conclusion

The study introduces 18 core criteria crucial for prioritizing integrated diagnosis interventions, offering valuable guidance to policymakers, funders, implementers, and manufacturers in LMICs. The overarching objective is to improve patient experiences and health outcomes. Emphasis is placed on ensuring that essential elements for success are thoroughly considered during intervention design. Moreover, rather than focusing narrowly on diagnostic tools alone, the study suggests the need for a more holistic approach—one that considers the full spectrum of patient needs and explores how diagnostic interventions might better align with and strengthen other components of the broader healthcare system.

## Supplementary Information

Supplementary Material 1.

Supplementary Material 2.

## Data Availability

All the necessary data is provided within the manuscript and supplementary files.

## Abbreviations

FIND: Foundation for Innovative New Diagnostics
HCW: Health care workers
HIV: Human immunodeficiency virus
LMIC: Low- and middle-income countries
M&E: Monitoring and Evaluation
NCD: Non communicable diseases
PHC: Primary Health Care
TB: Tuberculosis
WHO: World Health Organisation
PEN: WHO's Package of Essential Non-communicable Disease Interventions
