# Supplementary material for: Criteria for designing integrated diagnosis interventions in low resource settings at the primary care level: a Delphi consensus study
Source: BMC Health Serv Res. 2025 Aug 25;25:1130. doi: 10.1186/s12913-025-13114-9 (PMC12379388; doi:10.1186/s12913-025-13114-9)
Supplement: Supplementary file 1 — Supplementary Material 1. [file 12913_2025_13114_MOESM1_ESM.pdf]

Annex 1: Questionnaire for the Delphi study

Page 1: Information Sheet

The research aims to develop the minimum criteria necessary when designing integrated diagnosis interventions at the primary care level in low-resource settings such as Africa. Integrated diagnosis allows for multiple conditions to be identified within one visit, and results are received by the patient on the same day. Integrated diagnosis can ensure early diagnosis of critical conditions, increase uptake of health services, and improve disease surveillance, patient experiences and health outcomes. Various integrated diagnosis interventions are implemented that differ by purpose, type of models, diseases combined, target populations, the scale of interventions, and results making it challenging to have clear evidence of what works effectively in specific contexts.

Establishing minimum criteria when developing integrated diagnosis interventions is helpful for implementers and manufacturers of diagnostic equipment to ensure they can achieve optimal patient experiences and health outcomes. Ultimately, this will allow systematic monitoring and evaluation of the interventions to ensure continuous learning and improvement. These criteria may change as new evidence emerges and our understanding of integrated diagnosis develops.

You have been selected to participate in the research because you have experience or knowledge in integrated health care or diagnosis as either a policy maker, implementer, health care worker, technical expert or academic, with experience and knowledge in primary health care in low- and middle-income countries (LMICs). Even though you may have been identified through your affiliation with a particular organization, your views will not be taken to represent those of the organization or government. You are engaged as an expert in your domain. We expect two survey rounds; a third may be needed if a consensus has not been reached or if there is consensus on everything and a prioritization exercise is needed.

Participating in this Delphi study is voluntary. You are free to withdraw from the survey at any time without giving any reason, by closing the tab in the browser or by sending an email to me at gamuchirai.gwaza@kellogg.ox.ac.uk. If you tick the 'no' option on the consent box, you will also be directed to a page that allows you to exit the survey. Withdrawal will not be possible after the data has been aggregated and analyzed.

We will follow data protection requirements for the UK GDPR and the Data Protection Act. The University of Oxford recommends the JISC platform, which will be used, and the data will be stored on the University of Oxford server. Following the principle of data minimization, we will only collect personal data needed for the research. You will be asked to indicate your age, place of work and profession by selecting from broad categories. No one will be able to identify you through your survey responses except for the researcher.

Whilst the researcher will take every care with the data you share, you should only take part in the study if you are prepared for your responses to be made public. This is because the online platform could theoretically be breached or hacked outside the researcher's control.

I confirm that I meet the inclusion criteria and consent to participate in this survey with a complete understanding of the aim and use of the results.

☐ Yes
☐ No

Demographics

The results will be aggregated and anonymized. These demographic details will be used to show the validity of opinions shared and the diversity of the experts that participated in the survey.

Sex *

Male	Female	Other
◯	◯	◯

Age*

18-25	26- 30	31- 40	41-50	51-64	64- and above
◯	◯	◯			

Role/Profession/Job title * (Tick all that apply)

Health Policymaker,	Academic Researcher/ Student	Professional working in a Global health organization, civil society organizations, NGOs	Health worker, e.g. physician, specialist, laboratory, biomedical specialist, scientist, nurse	Lay health worker, community health worker, and other work within a health facility	64- and above
◯	◯	◯			

Country where you are based *

____

Country (ies) where you have experience and knowledge of the health system *

____

____

____

____

____

Category 1: Governance and Leadership

How important is it (for effective implementation that improves patient experiences and health outcomes) to have the following as minimum criteria when developing an integrated diagnosis intervention for primary care facilities?

1. **Clear and specific funding for the diagnosis and treatment of targeted diseases or conditions**

Not important	Limited importance	Important but not critical	Critical to include	Unable to rate (not my expertise)
1	2	3	4	5
◯	◯	◯	◯	◯

2. **Funding for continued training of the health workers for diagnosis of the targeted conditions**

Not important	Limited importance	Important but not critical	Critical to include	Unable to rate (not my expertise)
1	2	3	4	5
◯	◯	◯	◯	◯

3. **A system to coordinate donor support to avoid disease fragmentation, if the funding for the different diseases/conditions is separate**

Not important	Limited importance	Important but not critical	Critical to include	Unable to rate (not my expertise)
1	2	3	4	5
◯	◯	◯	◯	◯

4. **A WHO policy or recommendation for the integration of the diseases/conditions**

Not important	Limited importance	Important but not critical	Critical to include	Unable to rate (not my expertise)
1	2	3	4	5
◯	◯	◯	◯	◯

5. **A national policy or guideline for the integration of the diseases/conditions** *

Not important	Limited importance	Important but not critical	Critical to include	Unable to rate (not my expertise)
1	2	3	4	5
◯	◯	◯	◯	◯

6. **A diagnostic algorithm or screening tool to guide the integrated diagnostic process**

Not important	Limited importance	Important but not critical	Critical to include	Unable to rate (not my expertise)
1	2	3	4	5
◯	◯	◯	◯	◯

7. **Treatment or clear referral pathway for treatment after diagnosis of all included diseases/conditions available.**

Not important	Limited importance	Important but not critical	Critical to include	Unable to rate (not my expertise)
1	2	3	4	5
◯	◯	◯	◯	◯

8. **Strong leadership, with a shared vision and support of integration at the central level as well as facility level**

Not important	Limited importance	Important but not critical	Critical to include	Unable to rate (not my expertise)
1	2	3	4	5
◯	◯	◯	◯	◯

Please provide any feedback on the wording of this criteria, suggestions for other thresholds or values of this criteria, and an explanation of your rating:

Category 2: Operational

How important are the following operational considerations at a primary care facility when developing an integrated diagnosis intervention?

9. **The complexity of diagnosis for the disease to be integrated** *

Not important	Limited importance	Important but not critical	Critical to include	Unable to rate (not my expertise)
1	2	3	4	5
◯	◯	◯	◯	◯

10. **All the diseases/conditions included in the integration have similar prevalence**

Not important	Limited importance	Important but not critical	Critical to include	Unable to rate (not my expertise)
1	2	3	4	5
◯	◯	◯	◯	◯

11. **Similar target population or risk profile of the intended beneficiaries of the interventions**

Not important	Limited importance	Important but not critical	Critical to include	Unable to rate (not my expertise)
1	2	3	4	5
◯	◯	◯	◯	◯

12. **Combined clinical and ICT systems, including a single registration form for patients to access all integrated services**

Not important	Limited importance	Important but not critical	Critical to include	Unable to rate (not my expertise)
1	2	3	4	5
◯	◯	◯	◯	◯

13. **Healthcare workers have specific training on patient-centred care** *

Not important	Limited importance	Important but not critical	Critical to include	Unable to rate (not my expertise)
1	2	3	4	5
◯	◯	◯	◯	◯

Please provide any feedback on the wording of this criteria, suggestions for other thresholds or values of this criteria, and an explanation of your rating:

Category 3: Facility Integration

How important is having the following criteria at a primary care facility when the integrated diagnosis is primarily based on the co-location of services or shared space across different rooms or buildings, with services provided by various health professionals?

14. **Sufficient physical space to conduct the medical examinations in private** *

Not important	Limited importance	Important but not critical	Critical to include	Unable to rate (not my expertise)
1	2	3	4	5
◯	◯	◯	◯	◯

15. **Sufficient and comfortable space at the health facility to sit while patients wait their turn**

Not important	Limited importance	Important but not critical	Critical to include	Unable to rate (not my expertise)
1	2	3	4	5
◯	◯	◯	◯	◯

16. Clear directions for patients on how to navigate the facility to access the different services *

Not important	Limited importance	Important but not critical	Critical to include	Unable to rate (not my expertise)
1	2	3	4	5
◯	◯	◯	◯	◯

17. **Training of the health workers in interpersonal collaboration** *

Not important	Limited importance	Important but not critical	Critical to include	Unable to rate (not my expertise)
1	2	3	4	5
◯	◯	◯	◯	◯

18. **Trust and respect among the health workers of each other's expertise** *

Not important	Limited importance	Important but not critical	Critical to include	Unable to rate (not my expertise)
1	2	3	4	5
◯	◯	◯	◯	◯

19. **Clear roles assigned to each health worker involved at the facility** *

Not important	Limited importance	Important but not critical	Critical to include	Unable to rate (not my expertise)
1	2	3	4	5
◯	◯	◯	◯	◯

20. **Functional referral mechanism to access the other services** *

Not important	Limited importance	Important but not critical	Critical to include	Unable to rate (not my expertise)
1	2	3	4	5
◯	◯	◯	◯	◯

21. **Follow-up mechanisms of patients as to whether they accessed all the required diagnostic services as well as necessary treatment**

Not important	Limited importance	Important but not critical	Critical to include	Unable to rate (not my expertise)
1	2	3	4	5
◯	◯	◯	◯	◯

Please provide any feedback on the wording of this criteria, suggestions for other thresholds or values of this criteria, and an explanation of your rating:

Category 4: Individual/Human Resource Integration

How important is having the following minimum criteria at a primary care facility when the integrated same-day diagnosis relies on service provision by the same health worker for all the targeted conditions?

22. Training in the diagnosis (i.e., full consultation and in conducting point-of-care testing) and follow-up counselling

Not important	Limited importance	Important but not critical	Critical to include	Unable to rate (not my expertise)
1	2	3	4	5
◯	◯	◯	◯	◯

23. The health worker should initiate the diagnosis rather than rely on the patient to request it.

Not important	Limited importance	Important but not critical	Critical to include	Unable to rate (not my expertise)
1	2	3	4	5
◯	◯	◯	◯	◯

24. A plan of how long it takes on average to conduct the diagnosis, i.e., full consultation and the tests

Not important	Limited importance	Important but not critical	Critical to include	Unable to rate (not my expertise)
1	2	3	4	5
◯	◯	◯	◯	◯

25. Continuity of care, one health worker manages the health care of the same patient.

Not important	Limited importance	Important but not critical	Critical to include	Unable to rate (not my expertise)
1	2	3	4	5
◯	◯	◯	◯	◯

Please provide any feedback on the wording of this criteria, suggestions for other thresholds or values of this criteria, and an explanation of your rating:

Category 5: Technology Integration

How important is each of the following criteria when the integrated diagnosis is based on a multiplex or multi-disease platform, e.g., GeneXpert or dual rapid diagnostic test (RDT)

26. All the conditions for the instrument to function are met, e.g., electricity and temperature control.

Not important	Limited importance	Important but not critical	Critical to include	Unable to rate (not my expertise)
1	2	3	4	5
◯	◯	◯	◯	◯

27. The test can be used as a screening tool, i.e., it may need a further test to confirm the results.

Not important	Limited importance	Important but not critical	Critical to include	Unable to rate (not my expertise)
1	2	3	4	5
◯	◯	◯	◯	◯

28. **The test should be both a screening and confirmatory tool for the results.** *

Not important	Limited importance	Important but not critical	Critical to include	Unable to rate (not my expertise)
1	2	3	4	5
◯	◯	◯	◯	◯

29. The health worker should be able to conduct the test rather than rely on other health workers to perform the test.

Not important	Limited importance	Important but not critical	Critical to include	Unable to rate (not my expertise)
1	2	3	4	5
◯	◯	◯	◯	◯

Please provide any feedback on the wording of this criteria, suggestions for other thresholds or values of this criteria, and an explanation of your rating:

Category 6: Monitoring and Evaluation (M&E)

How important is it that the following minimum conditions are met when there is an integrated diagnosis intervention of any type, i.e. individual, facility or technology integration?

30. An M&E framework with measurable indicators of targeted patient satisfaction and health outcomes

Not important	Limited importance	Important but not critical	Critical to include	Unable to rate (not my expertise)
1	2	3	4	5
◯	◯	◯	◯	◯

31. A requirement for health workers to provide reports on all the diseases/conditions being integrated either to the donor or government system

Not important	Limited importance	Important but not critical	Critical to include	Unable to rate (not my expertise)
1	2	3	4	5
◯	◯	◯	◯	◯

32. Single reporting form or tool at the facility for all targeted conditions/diseases

Not important	Limited importance	Important but not critical	Critical to include	Unable to rate (not my expertise)
1	2	3	4	5
◯	◯	◯	◯	◯

33. **Customer feedback mechanism to report on the quality of service** *

Not important	Limited importance	Important but not critical	Critical to include	Unable to rate (not my expertise)
1	2	3	4	5
◯	◯	◯	◯	◯

Please provide any feedback on the wording of this criteria, suggestions for other thresholds or values of this criteria, and an explanation of your rating:

THANK YOU

Your contribution to research is highly appreciated. This is the first round, and once the results have been analyzed, a second round of the survey will be conducted within the next 6 to 8 months for areas where consensus still needs to be reached. Please feel free to reach out with any more feedback or thoughts on the topic.
