# Supplementary material for: Criteria for designing integrated diagnosis interventions in low resource settings at the primary care level: a Delphi consensus study
Source: BMC Health Serv Res. 2025 Aug 25;25:1130. doi: 10.1186/s12913-025-13114-9 (PMC12379388; doi:10.1186/s12913-025-13114-9)
Supplement: Supplementary file 2 — Supplementary Material 2. [file 12913_2025_13114_MOESM2_ESM.pdf]

SOCIAL SCIENCES & HUMANITIES
INTERDIVISIONAL RESEARCH ETHICS COMMITTEE
DEPARTMENTAL RESEARCH ETHICS COMMITTEE

DREC for the Department for Continuing Education. researchethics@conted.ox.ac.uk

8 August 2023

Dear Pamela,

Research ethics approval

Research title: A Delphi study to set minimum criteria for developing integrated same-day diagnosis interventions in LMICs

Research ethics reference: OUDCE C1A 23 033

The above application has been considered on behalf of the Department for Continuing Education Departmental Research Ethics Committee (DREC) in accordance with the University's procedures for ethical approval of all research involving human participants.

I am pleased to confirm that, on the basis of the information provided to the DREC, ethics approval has now been granted for this study.

Please note the following:

Personal data: It is the responsibility of the PI to ensure that all personal data collected during the project is managed in accordance with the University's guidance and legal requirements.

In-person activities: Any data collection involving in-person interactions with participants must have an up-to-date fieldwork risk assessment in place; further guidance is available from the Safety Office's website.

Amendments: Please notify the committee if you intend to make any amendments to the information in your ethics application as submitted at date of this approval, as all changes must receive ethical approval prior to implementation. The amendment form is available on the SSH IDREC webpage.

We welcome feedback on your experience of the ethical review process and suggestions for improvement. Please email any comments to researchethics@conted.ox.ac.uk or ethics@socsci.ox.ac.uk.

Yours sincerely

Matthew Weait

DREC Chair

cc: Annette Pluddemann, PI
